# “Welcome to the Revolution”: Promoting Generational Renewal in Argentina’s Ni Una Menos

**DOI:** 10.1007/s11133-023-09530-0

**Published:** 2023-02-20

**Authors:** Elisabeth Jay Friedman, Ana Laura Rodríguez Gustá

**Affiliations:** 1 Politics Department, University of San Francisco, San Francisco, CA 94117 USA; 2 Instituto de Investigaciones Políticas UNSAM - CONICET, Escuela de Política y Gobierno, Universidad Nacional de General San Martín, UNSAM Campus Miguelete, 25 de Mayo y Francia, San Martín, Provincia de Buenos Aires C.P.: 1650 Argentina

**Keywords:** Generational renewal, Feminist generations, Feminist activism, Social movements, Youth mobilization, Ni Una Menos, Argentina

## Abstract

Despite the global upsurge of youth-fueled mass mobilization, the critical question of why new generations may be eager to join established movements is under-explored theoretically and empirically. This study contributes to theories of feminist generational renewal in particular. We examine the longer-term movement context and more proximate strategies that have enabled young women to participate steadily in a cycle of protest, alongside more seasoned activists, due to a process of feminist learning and affective bonding that we call “productive mediation.” We focus on the Argentine Ni Una Menos (Not One Less) massive yearly march, which, since its onset in 2015, demonstrates that feminist activists have achieved the sought-after goal of fostering a highly diverse mass movement. These large-scale mobilizations against feminicide and gender-based violence gain much of their energy from a strong youth contingent, so much so that they have been called “the Daughters’ Revolution.” We show that these “daughters” have been welcomed by previous generations of feminist changemakers. Drawing on original qualitative research featuring 63 in-depth interviews with activists of different ages, backgrounds, and locations across Argentina, we find that long-standing movement spaces and brokers, as well as innovative frameworks of understanding, repertoires of action, and organizational approaches, help to explain why preexisting social movements may be attractive for young participants.

## Introduction

On June 3rd, 2015, the Ni Una Menos (Not One Less - NUM) massive yearly march erupted across Argentina to protest the feminicide of young women. In the capital, Buenos Aires, more than 300,000 people gathered to march and chant before taking part in an enormous rally outside the Congressional Palace. There, feminist artists and journalists read a “Manifesto” demanding a robust state response to gender-based violence. It was the largest such march in Argentina’s history, and instantly spread from Mexico to Uruguay, eventually even reaching South Korea and Poland. It sparked a cycle of mass protest within and beyond Latin America, including the 2017 and 2018 Global Women’s Strikes on International Women’s Day. By 2018, NUM had been replicated in at least six hundred cities across the world (Castro 2018); in Argentina, mass activism expanded to the abortion rights mobilizations that lasted until Congress legalized the procedure in December 2020.

With NUM, Argentine feminism achieved a central goal of many progressive social movements: a highly diverse, mass base anchored in a robust social network permeating all types of social groups in a range of locations. A NUM founder described the broad social penetration of NUM, whose flexible and amoeba-like shape pushed the boundaries of more traditional movement organization:

I think it is difficult to characterize Ni Una Menos, there are several overlapping spaces, like mounting one on top of another. … Many Ni Una Menos [groups] came from various places; there are Ni Una Menos in the provinces … the Ni Una Menos movement [is] one that includes many diverse actors … Afterwards, everything that Ni Una Menos proposed was taken up by a lot of actors and not only by the [founding] collective. (NUM organizer 1, LATFEM feminist journalist network)

One of its most striking features was the unprecedented integration of thousands of young and very young women,^1^ who participated with their groups, friends, family, or on their own. Marching under homemade banners and signs, their bodies, plastered with slogans, shone with bright paint and glitter. The so-called “Daughters’ Revolution” took experienced activists by surprise – and inspired them. As one feminist with a long trajectory of movement activism in multiple sites expressed:

My feeling is that one has rowed against the tide for a long time and suddenly we are being pushed by a sweeping wind, which sweeps us into good health. It surprises me, it pushes me to a greater radicalism in many things, where I was more prudent, and I see that young women and teenagers are … taking other risks because fear is lost. (Experienced feminist 1, multisited activist)

As this testimonial indicates, fearless younger participants pushed older feminists, and feminism, forward.

NUM co-founder and scholar-activist Maria Pia Lopez argues that, beyond Argentina, “feminism has become the largest source of youth activism” in Latin America (López 2020, 68). NUM was preceded by other youth-fueled mass feminist activism. The Marchas de Putas/Vadias – translations of the transnational “Slut Walk”– brought together young women, self-identified transfeminists, and sex workers to protest gendered violence in the early 2010s. During Brazil’s 2015 “feminist Spring,” peasant women, Afrodescendant women, and students rejected sexual assault and advanced overlapping agendas (Snyder and Wolff 2019, 96 − 7). Beyond swelling NUM’s ranks at home and abroad and providing the rank and file of the Women’s Strikes, youth-driven protest has reverberated in viral protests such as the powerful 2019 performance “Un Violador en Tu Camino” (“A Rapist in Your Path”) invented by Chile’s Las Tesis collective.

Latin American feminist activism illustrates what initially may seem to be a generational shift, part of what has been designated as a “fourth wave” of global feminist activity, with younger activists taking the lead in contemporary feminist and democratic protests in the North and South (Cochrane 2013; Chamberlain 2017; Molyneux et al. 2020; Natalucci and Rey 2018; Ponce Lara 2020). But, as reflected in other regions and activisms, the NUM movement exhibits what might be more accurately described as a renewal: the rejuvenation of a movement that still includes earlier generations. Such rejuvenation is remarkable given that intersectional tensions, among which generational differences have loomed large, have characterized this movement arena. Why was NUM able to overcome this long-term challenge to feminist movement building in Latin America to create a dynamic space where generations could come together? The answer to this question speaks to the more general concern with the conditions necessary for generational renewal within movements.

To understand why NUM was able to break a history of intergenerational distrust and foster mass mobilization, we first situate our work within previous research on generational analysis and social movements, focusing on youth and (regional) feminist activism. With this case study, we also add to the blossoming literature on Latin American feminist intergenerational cooperation by bringing it into dialogue with the insights drawn from more general scholarship on micromobilization, or movement recruitment.

We then turn to empirical analysis of NUM, based on original interviews with 63 organizers and participants from different age cohorts, as well as data from contemporary primary sources. On the one hand, we find that in ways both deliberate and organic, previous generations of feminists established spaces and networks, and offered insights, through which younger participants developed movement identities and solidarities. On the other hand, NUM organizers appealed to a younger generation by replacing rationally bounded, adult-centered claims with emancipatory frames, and advancing fluid structures that made room for performative repertoires. The result was a new generation of activists seizing the opportunity to engage in digitally connected and face-to-face mass activism against gender violence.

## Explaining Social Movement Rejuvenation

With the definitive protagonism of young, and sometimes very young, activists in the cycle of mass demonstrations beginning in 2011’s “Arab Spring” and reaching a peak in the 2019 Climate Strike, social movement analysts are paying more attention to the importance of age, and more specifically, generation, to mobilization and social protest. While some of these protests have been youth-driven, others manifest long histories and contemporary instances of intergenerational cooperation. Understanding why some social movements are attractive for youth, and young women in particular, requires us to consider generational transformation beyond simple demographic transition from older to younger participants. With this in mind, we seek to address if and how social movements can bridge generational distances given that younger cohorts always bring challenges in terms of protest styles, including frameworks of understanding, forms of organizing, and types of actions.

### Generational Styles in the 21st Century

Truly intergenerational movements face a considerable hurdle: how to incorporate distinct political generations with often very different styles of sense-making, organizing, and activism. As we consider the context in which Latin American feminism has developed, it does seem as if the 21st century upsurge of youth activism, and NUM specifically, reflects a new political style.

But before turning to what this new style entails, we need to clarify what Karl Mannheim, the influential political generation theorist of the 20th century, defined as a “generational unit.” He argued that political generations, rather than being the result of lifecycle phenomena attached to biological age, are constructed through young people’s shared experiences of major historical events, particularly the “dynamic de-stabilization” of rapid sociocultural change (Mannheim 1952, 303). People who come of age in times of social, economic, and/or political upheaval will be marked by those experiences. As such, political generations emerge through the shared experiences, ideologies and identities forged in a particular context (Cullen and Fischer 2014; Molyneux et al. 2020). As Mannheim (1952) cautioned, not all young people are affected in the same way; for example, they might respond in more liberal or conservative directions, forming distinct “generational units” (304). Without ignoring the impact of “forerunners” from earlier generations, and the fact that age and cohort may not be perfectly aligned (Borland 2014; Mannheim 1952, 308), such generational units become influential when they capture the *zeitgeist* of the time.

According to Mannheim, what attests to the existence of a new generational unit is “the formation of a new generational style” made up of elements such as “new conceptions,” undergirded by “psychological and intellectual impulses,” leading to “formative principles,” and resulting in “social and intellectual currents” (Mannheim 1952, 306–315). Research on activist generations reveals that such a style perdures over time. Those mobilized during the height of contentious politics are the most likely to continue to identify or embody those values later on (Caren et al. 2011; Whittier 1997). Generations matter to activism.

In Latin America and elsewhere, in the face of an uncertain and destabilizing future – subject to climate crisis, economic inequality, precarity in education, employment and social protections, within a hybrid reality where social media are integrated into every aspect of economic, social, and political life – a progressive youth movement style has materialized. Drawing together insights from research on the last decade’s youth activism, we characterize this style in terms of its complex understandings of multiple inequalities, highly flexible organizations embedded in digital resources, and forward-looking prefigurative repertoires (della Porta 2019a, 1416; Juris and Pleyers 2009; Molyneux et al. 2020; Sutton 2020). We summarize this style in Table 1 and explain it below.

**Table 1:** Progressive youth movement style

Movement element	Progressive youth style
Framing	Emancipatory Radical intersectionality as core frame to capture identities and inequalities
Organizing processes	Networks of purpose; fluid structures and boundaries Horizontal ties On-line and off-line worlds inextricably linked
Repertoires	Prefigurative “Politics of the street”; transformation rooted in cultural actions Discursive and performative centrality of the body

Source: own elaboration

#### Emancipatory Frameworks Against Multiple Oppressions

Younger activists tend to be more accepting of a range of differences from the “norm” or privileged subject, in terms of ethno-racial or gender identity, sexuality, citizenship status, and the like (Chironi 2019; della Porta 2019b; Portos 2019). Young women politicize personal experiences, a well-grounded approach in Latin America (Safa 1990) that transcends an individualistic focus to link to intersecting oppressions (Larrondo and Ponce Lara 2019). They are also connected to the youth politics around them, whether bringing their perspectives into youth spaces and student movements, or those focused on the environment, ethnicity, sexual and gender identity, and economic justice. For example, Jessica Taft’s 2011 study of teen girl activism in Vancouver, San Francisco, Buenos Aires, Mexico City, and Caracas shows it to manifest a worldview without prejudice or hierarchy, inclusive and ecological, “informed by the anti-oppression analyses of feminism, anti-racism, and other radical ideologies of difference and power” (159). These activists’ frameworks of understanding recognize and take as fundamental the intersectionality of multiple oppressions, and the need to address them simultaneously.

#### Networks of Purpose and Internet Incorporation

Organizationally, young activists prefer horizontal “networks of purpose” that may not last longer than a particular campaign, anchored by active involvement and personal commitment (Coe and Vandegrift 2015; Juris and Pleyers 2009; Portos 2019). At the transnational level, Jeffrey S. Juris and Geoffrey Players find, “alter-activists” have focused on participatory, egalitarian organizing and “are committed to an ethic of openness, local-global networking, and organising across diversity and difference” (Juris and Pleyers 2009, 63). Critically, such value transmission frequently takes place through peer socialization. Even the youngest activists invest in creating inclusive and accessible spaces to develop and transmit their own ideas (Gordon and Taft 2011, 1519).

Every aspect of this youth movement style incorporates the internet, to the point where it is unproductive to differentiate between on and offline arenas (Maher and Earl 2019, 868). Fundamentally, youth interactions and connections firmly embed the internet, which also weaves through their activist cultures and actions (Coe and Vandegrift 2015, 139; Maher and Earl 2019, 872). As Thomas V. Maher and Jennifer Earl find, it can both “augment traditional paths to activism and offer new avenues for participation” with familiar and new people and sources (Maher and Earl 2019, 877). As a result, even more than for older generations, “online networks” enable young people’s emergence as activists (Crossley 2017, 158). Rossana Reguillo Cruz argues that “technologically mediated contact” produces affective “zones of intensity” that can bind participants to the cause and each other, regardless of their physical location (Reguillo Cruz 2017, 144). Creative, performative direct action rapidly diffuses via social media, as activists replicate or translate it into the vernaculars of local struggles (Juris and Pleyers 2009), contributing both to collective identification and subsequent mobilization (della Porta 2019a, 1415).

#### Prefigurative Politics and Creative Repertoires

In capturing a generational style, researchers argue that youth often demonstrate prefigurative politics, that is, “attempting to construct the future in the present, rather than postponing it to after the achievement of state power” (della Porta 2019a, 1416). Nowhere is that more evident than in young activists’ repertoires, which privilege “artistic, cultural, and ludic activities” to create accessible awareness and build communities where they have a pleasurable sense of belonging (Coe and Vandegrift 2015; Chironi 2019; della Porta 2019b, 1586). For example, activist girls in the Americas “build a wide range of communities and institutions that reflect their ideals of democracy, equality, diversity, justice, freedom, and solidarity…[in] a hopeful and utopian mode of activism” (Taft 2011, 162). They focus on public education through a host of creative approaches from murals to theater, seeking to make participation enjoyable, democratic, and egalitarian. They center performative engagement, with a profound focus on the “territory” of the body. Although this focus is far from new, Marina Larrondo and Camila Ponce Lara argue that younger activists take the “the body as axis of their political action,” making it a central “tool” of protest; an “instrument of visibility” uniting knowledge and power (Larrondo and Ponce Lara 2019, 13–15).

Taken together, contemporary movement developments indicate the emergence of a new progressive youth style of protest. Given these trends among young women’s organizing, the creation of a truly intergenerational feminist movement requires conditions that enable or even encourage the incorporation of this style into the movement at large. But, as revealed by previous work on the impact of generational differences in feminist movements, those conditions are not easily met or may fail to accommodate younger styles of activism. Intergenerational dialogue and cooperation can make for complex movement politics.

### Generational Renewal: From Disidentification to Productive Mediation

Manifesting their own style embedded within the problems and experiences of their times, new generations who are inspired to join pre-established movements may seek to alter movements’ strategies, tactics, political culture, and organizational structures (Whittier 1997, 774). Consequently, young people “do not merely add cultural aspects to existing political forms,” but may well transform the shape of the movement (Coe and Vandegrift 2015, 142). Such generational encounters offer the potential for significant discord, but also can bring about opportunities for cooperation. Far from a uniform process, generational interactions can take on the different forms we explain below, which range from irreconcilable conflicts to truly intergenerational collective actions and feminist revival.

#### Disidentification

The difficulties of fostering intergenerational movements are well-documented in past and present movements. Considering feminist movements in the US, Italy and Spain, Whittier (1995), Chironi (2019), and Portos (2019) explain conflictual dynamics as based on different generations’ identities, ideologies, strategies, and tactics, or what could be seen as distinct generational styles. In the US, such conflict led Henry (2004) to coin the concept of “disidentification” between so-called “third” and “second” wave feminists. Younger women insisted that they “were not their mothers’ sisters,” as they sought to differentiate their feminism. As Whittier (2004) affirmed, “third wave” feminists “define[d] a collective identity … distinguished largely by its attempts to depart from second-wave feminist collective identity despite similarities in ideology and goals” (538; see also Mann and Huffman 2005). Today, some analysts find that younger women’s mobilization continues to conflict with more seasoned feminists’ perspectives, particularly with respect to its radicality and sustainability (Lamas 2021). Such disidentification does not easily lend itself to intergenerational mass mobilization, and instead may lead to a failure of cooperation that fragments the movement.

As alluded to above, such findings are reflected in, and perhaps reinforced by, the common characterization of women’s movements experiencing generational replacement – rather than renewal – by means of movement “waves.” Because waves are understood as the collective actions of discrete generational units, rather than temporal oscillations within and/or across generations, this concept tends to echo the assumption that new generational units will be more likely to replace (and even displace), rather than join, older cohorts. Analytically, the much-used “wave” metaphor can reduce the transformation and growth of women’s movements into a sequential order of distinct cohorts with irreconcilable generational styles. However adequate in capturing the most conflictive aspects of feminist movements, the metaphor has been widely critiqued due to its restriction of the scope and space, and thus actors and actions, that do not fit neatly into the chronological narrative (Hewitt 2010; MacLeavy et al. 2021). Other aqueous metaphors may be more apt: for example, distinct forms of collective action “that flow and branch over time” would be better captured as a river (Goss 2013, 14). The mass, multigenerational pro-abortion movements in Latin America that were inspired by Ni Una Menos’ approach are known as the *marea verde* or “green tide.” The application of the wave concept can obscure the trust building and cooperation among feminists from different generational units whose collective action has flooded the streets and squares of the Latin American region.

#### Spin-off

From another perspective, the seemingly intractable generational divide can be interpreted as an ongoing multigenerational movement with distinct cohorts coming to the fore at different times in different ways. This perspective accommodates some, albeit limited, forms of intergenerational cooperation. In Ecuador and Peru, for example, Anna-Britt Coe (2020) found that an older generation was keen on recruiting younger members, but in ways that ultimately led to the development of separate and distinct kinds of “spin-off” activisms (see also Borland 2014; Masson 2007). Through what the author calls “in/exclusionary practices,” professionalized feminist organizations brought in younger activists to participate in trainings, interventions, volunteer work, employment, and university gender studies. However, young adults were simultaneously excluded through organizational practices that subordinated their agency and goals. Chafing at age and bureaucratic hierarchies in these organizations, younger participants turned to generating their own spaces, structures, and practices of feminism. As a result, the younger generation was able to make use of experiences gained within an older women’s movement. However, they did so by establishing “new forms of activism” emphasizing horizontality and autonomy, in separate spheres of collective action.

Though richer in terms of the empirical phenomena it may help to explain, the concept of spin-off focuses on ideational and organizational inheritance, rather than cooperation. As a multigenerational effort, the women’s movement “allows for criticisms by the next generation to be viewed as an accomplishment in that … [an older] feminist generation has been successful in shaping the society and the discourse giving young women something to build upon and reject” (Reger 2012, 193; see also Cullen and Fischer 2014). With regards to contemporary, so-called “fourth wave” mobilizations, some find generational continuity in the themes of feminist mobilization and struggle, with departures in strategies and tactics (Molyneux et al. 2020). But generations’ struggles are still seen as distinct from one another rather than in fruitful coexistence, with this most recent “wave” characterized as the “online feminism” of younger participants. Although the concept of spinoff suggests that ties may not be dramatically severed between generations, it still underestimates deeper generational alliances for social protest and meaning making.

#### Productive Mediation

Achieving intergenerational mass feminist mobilization is no easy feat; as the discussion above makes clear, incorporating a new generational style is challenging, and generational divisions seem, or are seen, to be more the rule than the exception. In order to “see” and understand the kind of intergenerational movements that may characterize the recent upsurge of feminist mobilization, we advance the concept of “productive mediation.” It is productive and well-networked feminist praxis based on both knowledge transmission and openness to, if not outright encouragement of, newer cohorts’ ideas, organizations, and repertoires. The wide diffusion of feminist ideas necessarily implies collective co-creation of understandings by means of intergenerational exchange and mutual learning.

We derive the elements of this analytic lens by bringing together insights from the social movement literature on the micromobilization responsible for movement recruitment with the emerging work on contemporary feminist movements. As a complex social process, productive mediation requires enabling factors: compelling frameworks, channeled through social networks that lead to the development of movement identities, and actions based upon them, in collective settings.

Eliciting new entrants’ interests and affinities through appealing frameworks of understanding is a fundamental step in movement recruitment. Such frameworks may appeal on a rational basis through processes of intellectual learning and argumentation, exemplified by the “rational/cognitive” approach (e.g., Snow et al. 1986). However, affective factors are also central; as the affect/emotions approach suggests, successful frameworks’ emotional resonance may ultimately provide the most powerful fuel for deep engagement (e.g. Goodwin et al. 2001; Jasper 1998, 2011).

Much of the micromobilization literature perceives movement recruits as having little movement experience - or even preexisting awareness or commitments. Such an assumption draws on the widely critiqued “deficit model” of youth participation (Osler and Starkey 2003), where young people are presumed to be empty vessels, “ignorant and uncaring by virtue of their age…unaware of their rights,” thus necessitating “adults to intervene and socialize them properly” (Gordon and Taft 2011, 1523). But fostering an intergenerational movement requires the accommodation of youth’s new styles in recruitment efforts, rather than adultist intervention (Gordon 2007). Such a need manifests most clearly in the negotiation and collective construction of frameworks of understanding. As Sutton (2020) demonstrates in her analysis of the intergenerational abortion rights movement in Argentina, feminist mobilization has benefitted from intellectual and organizing processes centered on multidirectional knowledge transmission. She highlights that in this movement, the term *sororidad* (sisterhood) conveyed “women’s political ability to stand up for each other, to have each other’s back, and to work across differences towards a common vision” (5). In this collective framework construction, the affective dimension becomes most apparent: bonds of care, affection and complicity are the glue that ties together activists across ages, cohorts, and generations.

Powerful frameworks prove fundamental to recruiting possible adherents; but the networks that act as conduits for such frameworks, particularly those within which people are already embedded, are equally important. Martin Portos’ study of the Spanish feminist movement, which has “the highest mobilizational capacity” in the country, confirms micromobilization’s emphasis on networks in collective settings: he finds local, as well as national spaces for encounter – including political networks, physical assemblies, organizational collectives, and institutionalized centers – crucial (2019). Verónica Gago, the participant, theorist, and chronicler of NUM and the Women’s Strike, elaborates that assemblies have enabled the development of “collective intelligence” (Gago 2019, 165–190).

Previous studies on mobilization distinguish among types of networks within distinct collective settings. Some are “informal interpersonal” or “everyday sociality” networks that can politicize collective identity by seeking to “attach…collective grievances to specific targets” (Ward 2016, 864). For younger participants, school-based and peer networks play a fundamental role in movement expansion, providing the infrastructure for the classic “biographical availability” of young people (Crossley 2008). Networking in school settings enables the frequent eruption of student-based movements. As Hirsch (1990, 245) explains, “loosely structured, face-to-face settings” become “havens [where] people can easily express concerns, become aware of common problems, and begin to question the legitimacy of institutions that deny them the means for resolving those problems.” In other words, such settings provide plenty of space for consciousness raising. Then, participating in protest, surrounded by others similarly willing to challenge authority and demand resolution to their problems, leads to a sense of “collective empowerment” particularly through the necessary “collective decisionmaking” on movement actions (Hirsch 1990, 245–256). These experiences teach people to have collective identities: together, they puzzle out how to take action, and together, they take it.^2^

Other studies of movement networks demonstrate that recruitment builds on preexisting social connections, whether familial, religious, or political, within which protest veterans play key roles (Crossley and Diani 2018, 155). In the case of intergenerational movements, both Portos (2019) and Sutton (2020) draw attention to a mediating, middle-aged generation acting as what we identify as “brokers” both inside and outside of university communities; their life experience, organizational skills, “proximity to and understanding of different age groups” enable them to “sustain networks” among other essential roles in protest coordination (Sutton 2020, 9–10). In short, successful intergenerational movements depend on both networks and networkers.

Active commitment to renewal provides the final factor in promoting intergenerational mobilization. Portos (2019) focuses on the “broker” generation’s dedication. Similarly, Gordon (2007) concludes that older generations have important strategic roles in supporting youth activism, particularly when providing resources, extending legitimation, and offering “bridges to social movement histories” (652). Sutton adds more nuance to feminist movements’ commitment processes. She identifies the mutual recognition among generational units of each others’ strengths, such as elders’ experience and youths’ energy as well as new repertoires and identities (Sutton 2020). Such commitment sustains collective action.

All these factors that make possible productive mediation and, therefore, the integration of young women in pre-existing social movements, are still under-researched. Our examination of why Ni Una Menos was able to expand to young activists despite past generational differences in the national movement provides further insights into the dynamics of intergenerational collaboration.

As the foregoing discussion of productive mediation attests, we are taking a different approach from other prominent explanations that focus exclusively or primarily on movement emergence. We are not asserting that, as such, these arguments are unimportant. In fact, the factors often discussed with regards to movement emergence – such as moral shock, ongoing grievances, or macro-level changes in the contexts that people are living through – absolutely matter for the birth of NUM. Scandalous and horrific murders of young women spurred strong reactions that sparked the original upsurge of the movement. Extremely long-lasting grievances over long-postponed state action on what many perceive as a pandemic of gender-based violence amplified demands. But what we are trying to explain here is why the feminist movement in Argentina was rejuvenated by attracting younger adherents who jumped on board and brought their new style into the movement.

## Methods and Data

This study’s analytical strategy consists of a single case study with heuristic purposes (George and Bennett 2005). Case studies offer empirical density and are useful for theory building (Ragin 1992). This approach makes it possible to identify complex causal processes through which preexisting movements and older generations make room for younger generations and their new styles. NUM is also a global exemplar empirical case of youth incorporation into feminism. Given its massive nature and its transnational contagion effect, studying NUM can provide causal clues regarding the role of intergenerational dynamics in the expansion of feminist (and other) movements beyond Argentina.

The main data collection technique was in-depth, open ended and semi-structured original interviews. This technique is particularly useful in the study of social movement recruitment and dynamics (della Porta 2014). Between 2018 and 2021, our research team conducted a total of 63 interviews with NUM activists who had been involved with at least one direct action – participating in an assembly, helping to prepare the demonstration, marching in a parade, and the like. We covered a wide range of activists in terms of age, site of activism, and biographical profile. Young women were also situated in different urban centers, including three of the four largest Argentine cities – Buenos Aires, Rosario, and Mendoza – and several towns, including San Martín, Morón, Merlo, Ituzaingó, and Tres de Febrero (Province of Buenos Aires); and Villa Constitución (Province of Santa Fe).

To study intergenerational alliances, we designed a multi-tiered sample with four distinct groups within the feminist movement (see Table 2, Interview Sample Profile). First, we interviewed four seasoned or experienced feminists whose well-known trajectory in the feminist movement from different sites of struggle provides them with an overall perspective of the movement evolution and the irruption of NUM. Second, we contacted the original NUM collective and interviewed eight of them. Members of these two groups were easy to identify because they comprise relatively well-delimited universes of activists. The third group, the brokers, were a methodological challenge because this middle-age generation of feminists is numerous and positioned across distinct activist sites. We adopted a strategy of theoretical sampling and selected them from the main sites: feminist NGOs and informal collectives, popular or grassroots movements of unemployed workers, universities, unions, political parties, and state institutions with gender portfolios. The fourth group is of young women who participated in the NUM cycle of protests. We oversampled student activists given the relevance of young and very young women from high schools and colleges at the 2015 demonstration, as pointed out by NUM organizers as well as more experienced feminists. In selecting young women, we used snowball sampling with the help of our student research assistants. Also, one of the researchers (who lives and works in Argentina) provided additional names for interviews, based on personal contacts, thus adding to activist variation.

**Table 2:** Interview sample profile

Numbers of interviews, by group	Sites of struggle	Relevant characteristics^a^
6 experienced feminists	• Abortion rights movement (2) • Feminist NGO (1) • State gender institutions and legislatures (2) • Multiple sites (1)	• Average age: 60 • Middle-class, highly educated • Diverse political affiliations
8 NUM organizers	• Feminist and LGBTQ NGOs, collectives (5) • Independent professionals (2) • Trade union (1)	• Average age: 36 (range 24 to 50) • Middle-class with diverse educational backgrounds • Diverse political affiliations
18 brokers	• Unemployed workers’ movements (2) • University (2) • Trade unions (3) • Legislatures (2) • State gender institutions and legislatures (4) • Feminist NGO and informal collectives (4) • Political parties (3)	• Average age: 38 (range 30 to 53) • Diverse social backgrounds, with prevalence of middle-class • Mostly left-wing and from progressive faction of Peronism
31 young activists	• Educational settings (high school student unions and universities) (15) • Feminist collectives and networks (7) • Political parties (5) • No specific site at the time of the 2015 NUM protest (3).	• Average age: 19 (age range 14–32), with about one third below 18 (13) • Diverse class backgrounds; predominantly middle class • Mostly left-wing and progressive faction of Peronism

^a^Age calculated at time of the 2015 NUM

Following a format of “process-oriented interview” which seeks to grasp how social processes unfold (Tavory 2020), we obtained information about the factors that led young women to join the protests, including the trajectories of collective action and the networks that inspired them to be part of organizing spaces and marches. For all four groups, we asked questions about their frameworks and identities, their networks and more relational contexts, their site(s) of activism, and their more specific actions in the NUM cycle of protests.

Our team interviewed the first three groups in person between 2018 and 2020. We interviewed young activists during 2020 and 2021 over Zoom, due to the mobility restrictions caused by the COVID-19 pandemic. In all cases, interviews were recorded with consent and verbatim transcripts used for analysis. We interpreted them using the grounded theory method, according to which data collection and analysis occurs vis-à-vis the categorization and construction of interpretations (Strauss and Corbin 2002). We complemented this evidence with participant observation in marches (2015 to 2017), visual evidence (Twitter, Facebook and Instagram posts) and documentary evidence (the NUM manifestos from 2015 to 2021). With this triangulation, we achieved saturation of the data within the main categories. As agreed to with the interviewees, we have kept their names anonymous. However, to show the perspectives and protagonism of each participant, we identify their quotations using their group and site of struggle at the time of the 2015 NUM.

In terms of our own positionality, we make our knowledge claims aware that both of us are brokers: researchers and professors who are active in fostering feminist learning communities. Sensitive to the fact that our positions in the status hierarchies associated both with our age and profession could influence younger activists’ responses, we worked with advanced undergraduate research assistants who conducted the interviews with their peers. We provided these researchers, chosen through a competitive process, with specific qualitative training and feedback on the rapport and the framing of the questions. We also consulted with them on their interpretation of the interview material.

## Intergenerational Mobilization in Ni Una Menos^3^

On March 16, 2015, when the body of yet another murdered teenager, 19-year-old Daiana García, was found stuffed into a garbage bag by the side of the road in the Province of Buenos Aires, a small group of journalists, artists, lawyers, and other professionals came together at National Library’s “Museo del Libro” for a reading marathon aimed at publicizing and making feminicides visible. García’s death was emotionally wrenching in part because it resembled the disappearance of human trafficking victim Florencia Pennachi ten years earlier; the journalists had been covering such news since that time (Pisetta 2019). This group took the name Ni Una Menos (NUM) and held its first public demonstration on March 26 in a public square in Buenos Aires.

Two months later, Chiara Páez, a pregnant 14-year-old from Santa Fe province, was found buried in her boyfriend’s grandparents’ backyard after being beaten to death. This horrific incident compelled NUM organizers to call for a mass demonstration in front of Congress. The group gathered at “Casa del Encuentro,” an NGO that had taken upon itself the systematic collection of feminicide cases due to the lack of official data, and together they became the organizing committee. They circulated the hashtag #NiUnaMenos, which took off across Facebook, Twitter, and Instagram, as popular artists and prominent politicians began to post pictures with it. More than 110 Facebook pages named “Ni Una Menos” and the name of a city coordinated on-line as well as local off-line actions in preparation for the national protest (Laudano 2018). On the day of the protest, as hundreds of thousands took to the streets across Argentina, the presidential palace and congress building were lit up in fuchsia– the color of NUM’s logo.

As a social protest, NUM was a tipping point. The mass mobilization inspired #NiUnaMenos to trend in Argentina and around the world (Laudano 2018). The large number of a diverse range of protestors was highlighted by our informants, who also employed metaphors of “explosion” and “ocean” to emphasize the magnitude of the crowds. They contrasted it favorably with other large-scale mobilizations, demonstrating its historic nature:

It was above our heads, none of us had a dimension of what we were doing. Everything was amazing. (NUM organizer 2, journalist affiliated with the Madres de Plaza de Mayo)^4^

I remember it as an ocean of people and I also remember seeing different people, they were not the usual activists; they were someone’s aunt and grandmother; multiple identities that questioned society as a whole. (Young activist 1, local network against gender violence in Morón, Province of Buenos Aires)

For many younger participants, NUM was their entry into feminist identity and action. As their comments reflect, the historically diverse and massive protest brought about a radical shift in their own lives in terms of their self-perception and place in society:

I think that Ni Una Menos opened doors, it was a before and after. That activists and non-activist women of different ages joined together was something historic. It is no coincidence that it keeps repeating itself, it even unites those of us who may not agree on all other issues … Ni Una Menos is like the hinge, it changed the girls of my generation, it marked us forever. After June 3, 2015, you are no longer the same, I celebrate not being the same. (Young activist 3, high school chapter of Peronist party, City of Buenos Aires)

Young activists already involved with feminism at the time of NUM acknowledged the social relevance of the march as a key moment for the advancement of feminism. In their own terms, NUM was a “turning point,” it had “tremendous social value,” thus pointing to its macrosocial relevance in expanding feminist ideals and identities:

It’s not that it marked me, but it was a turning point for massification, and people who were not in the feminist movement began to ask some questions, women began to identify violence and the typical issues of feminism. (Young activist 5, local feminist informal collective “Feminismos del Oeste” [Feminisms of the West], Province of Buenos Aires)

It seems to me that it was of tremendous social value putting into public discussion issues that were covered up, silenced. It allowed the power of the movement, of the youth, to show … Personally, for me, it was not a pivotal moment, but I do recognize the value to the history of Argentine feminism cannot be questioned. (Young activist 6, university student union, Merlo, Province of Buenos Aires)

In the same vein, an older generation, while aware of a longer history of feminist action, recognized that NUM was an emotionally charged foundational moment for feminist youth:

Some very young women have said that feminism started with the Ni Una Menos march in 2015. I think what that reveals is that certain initiation experiences are something very powerful, when you became a feminist, when you saw what feminism was and even timidly, even without an adhesion, you noticed that there was something that could challenge and could represent you.(Experienced feminist 1, multisited)

Given the high salience of generational identity in Argentine protest politics, such as the well-known mothers and grandmothers human rights groups, the allusion to familial relations has an important contextual resonance. But it also intimates a sense of deep connection among the generations. Older activists and the media called these young women *las pibas* (Argentine slang for girls) in interviews and press coverage, a term highlighting that the march was crowded with not only young women but also high-school girls. These more experienced observers called NUM “the daughter’s revolution” or even “the granddaughters’ revolution.” But the revolutionaries came from multiple generations. The “revolution” led by *las pibas*, as the following quotations illustrate, produced changes in ideas and worldviews that, in turn, stemmed from other movement struggles and traditions.

[NUM] was a revolution of the minds, with the youth at the forefront. … It is true that there is dispute, but it is a revolution and when there is a revolution minds open, debates open, organizations appear … young women are the recipients of the revolution. (Experienced feminist 2, left-wing political party and national legislature)

Never has so much progress been achieved at the level of discussion, at social consensus; we have achieved a generational transmission and a massiveness that was built by hand and little by little. There I return to legacies, the legacy of the Mothers of Plaza de Mayo, of the Grandmothers. … I think the most important achievement is getting out, losing fear, being supportive among us, that question of ‘I believe you.’ (Broker 2, center-left political party and local executive, Morón, Province of Buenos Aires)

Because the march was truly intergenerational, interviewees extended the term revolution to all women, while placing young women in a privileged place. This rhetorical move wove together genealogy and legacy at the center of NUM, with a sense of feminist parenthood and sorority bonding the participants.

This diverse and massive multigenerational presence was surprising: opening the feminist field to a younger generation was far from a given in Argentina. Manzano (2019) elaborates on feminist movements’ historic tensions around renewal. Early feminists in the 1920s criticized the sociocultural and especially sexual mores of “modern girls.” In the 1960s, the hyper-sexualization and consumption-orientation of “liberated girls” resulted in older activists seeing them as victimized by or complicit with patriarchy and capitalism. And during the democratization of the 1980s, emerging “feminist groups constructed an agenda of demands and outlined a subject centered in the adult woman, preferably a mother” even as they challenged women’s restriction to their traditional roles (Manzano 2019, 54). By the end of the 1990s, long-time activists became troubled at their failure to recruit younger activists to the cause, to the point that they saw themselves as “dinosaurs” (presumably, not only because they were old, but also might go extinct) (Borland 2014). Self-identified feminists in Argentina did not center the needs of young women – for example, although abortion might well be a need of young women, it was characterized in adult-oriented terms – thus alienating or sidelining them (Manzano 2019). Overall, as Elizabeth Borland summarizes, a “lack of understanding, ‘generosity,’ recognition, and respect hurt the movement” (Borland 2014, 98).

Why was NUM able to dramatically shift this context and foster intergenerational mobilization? In more conceptual terms, what were the enabling conditions behind intergenerational productive mediation? The remainder of this study addresses these questions.

## Explaining Generational Convergence

Our empirical findings show three major factors that enabled generational convergence through ties of empathy and sorority as well as feminist learning and exchange. Enthusiastic participants overcame previous generational and other socially relevant divisions to engage in compelling social action due to: (1) collective spaces for meeting and interaction; (2) active mentorship of “brokers,” an in-between feminist generation with a significant capacity for sustained network building; and (3) social protest in many ways structured to resonate with younger people’s mobilizational style. The first two factors can be conceptualized as more structural and long-term conditions that provided the basic scaffold for NUM massification. The catalytic NUM collective enabled more proximate conditions by providing frameworks, forms of organizing and protest that fit with a progressive youth style.

### Feminist Foundations and Multisite Brokers

#### National Women’s Encounters

One of the most significant foundations for intergenerational alliances were the Encuentro Nacional de Mujeres (National Women’s Encounters, ENM) that offered fertile ground in which NUM germinated. The ENM hosted successive generations of women: “I believe that the continuity, massiveness and support of the National Women’s Encounters is something unique in the world. This has been achieved by the consistency and massiveness and effort of the women’s movement” (experienced feminist 3, abortion rights campaign). The ENM has exerted a powerful socializing effect on various generations. Initiated by experienced feminists, these meetings have been an entry door to feminism for brokers and, more recently, young women and teenagers.

A near-yearly meeting begun in 1986, the ENM is “an inspirational venue for learning, exchange, and support.” It moves around the country, with regional volunteer committees organizing a program where participants come together in a horizontal network, on a non-partisan basis, to take part in workshops and cultural activities, as well as an energetic public parade and demonstration (Friedman 2017, 133). The ENM gather together a diverse public to discuss a wide range of topics in workshops for a few days (Alma and Lorenzo 2013; Sutton 2020). One of its many benefits for building feminist communities around the country and beyond is the space it opens for inclusive activist networking, based on an intense process of development and organization (Masson 2007).^5^

If NUM catalyzed young activism, the ENM provided the spark for those who would become NUM’s brokers during the “second great depression” of the early 2000s. This deep political and economic crisis led to social upheaval known as the “Argentinazo,” that included the mobilization of the urban poor. This earlier generation took part in the society-wide protests against the deleterious effects of Argentina’s neoliberal transformation, and the political parties over being it. Organizing through the ENM resulted in demonstrations incorporating both low-income women in class-based social movements of unemployed workers (Borland and Sutton 2007; Di Marco 2011; Tabbush and Caminotti 2015), as well as women from left-wing political parties and the more progressive factions of Peronism. Some of the brokers recalled the powerful amplifying effects, as flows of women into the ENM then extended feminist ideas into other social movements.

In the post Argentinazo, there was a massive eruption of women from the popular assemblies, from the neighborhood [soup kitchens], women who were neither academics nor faculty activists, but had fed their families and communities [*mantiendo la olla*, literally “maintained the pot”] in the 2001 crisis. They came with accumulated experience … And all those women turned to the ENM and … it overflowed. I think that was the first moment of massiveness of the ENM. (Broker 1, university teachers´ trade union, City of Buenos Aires)

A turning point was my participation in the National Women’s Encounter of Mar del Plata, in 2005 with a colleague from the MAS [left-wing political party], just out of curiosity. …. It blew our minds; a lot of ideas began to dawn on us … I began to see the need to introduce the debate in my organization, which is the Socialist Left, and in different spaces, in that moment in the student center and after I graduated in the union, as well as in different social spaces, organizing the 8M [International Women’s Day demonstration] and mobilizing for other issues. (Broker 3, abortion rights campaign at her university, City of Buenos Aires)

As these quotations reflect, ENM participants adopted feminist or feminist-adjacent identities, becoming the brokers of future generations. For middle-class, educated women in university settings, the ENM were also central. Some of them organized their own intergenerational feminist groups, and even became hosts for the ENM in their provinces, such as Las Juanas’ coordination of the 2004 ENM in Mendoza. Significantly, activists at an ENM founded the Campaña Nacional por el Derecho al Aborto Legal, Seguro y Gratuito (National Campaign for the Right to Legal, Safe and Free Abortion), a truly intergenerational network, with seasoned feminists, middle-aged brokers, and young and very women, thus creating a flexible structure of territorial scope with abortion demands linked to freedom and self-determination (Burton 2019; Gutiérrez 2021).

More recently, younger women have also found the ENM a place to develop their feminist identities, clearly swelling its ranks. In the interviews, they described being invited by fellow youth activists, sometimes from leftwing parties, to an Encounter, “where everything started.” From this experience, they “fell in love” with feminism and had their first movement action, marching out with their friends at the end of the meeting, shouting at counterprotestors and beating the *bombo*, the large traditional drum omnipresent in Argentine protests.

These processes of gathering, debating, and marching together enabled and strengthened ties of empathy and solidarity among women from different generational units, as well as social, political, and institutional sites. The impact of the ENM was so substantial in young women’s imaginary that, when asked about feminist leadership or “referents,” many could not provide specific names but invariably said something similar to: “We cannot think of the women’s movement without the Encuentros that have happened for 35 years and make us discuss, for at least one weekend in Argentina, what we women need” (young feminist 7, university student union, Rosario, Province of Santa Fe). The ENM serves as the Argentine feminist movement keystone.

As a fruitful structural condition for productive mediation, the ENM had amplifying effects, as the emotional and intellectual experience of these intense meetings helped young women create and/or expand their own networks and groups. Wrapping the green scarf which is the now-regional symbol of the abortion rights movement around wrists and the straps of their shoulder bags, some returned to their left parties and student organizations and founded internal “women’s fronts” that in turn would become spaces for NUM organizing.

**Fig. 1:**
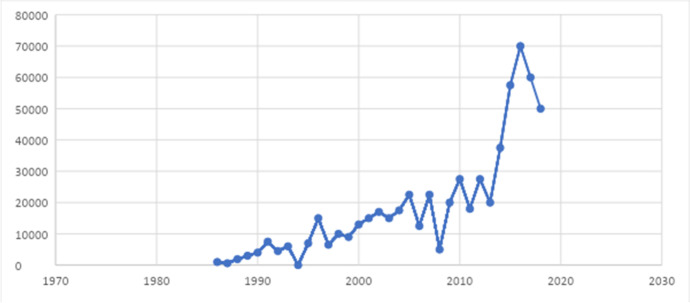
Total number of participants in ENMs, 1986–2019. Sources: based on data drawn from Alcaraz and Paz Frontera (2018) and Santoro (2019)

In a telling statistic, just one year before NUM, in 2014, the ENM had risen to 37,500 participants, an 87% increase relative to 2013’s 20,000 attendees. This significant expansion reflects a growing interest in feminist movement politics. After the first NUM, the 2015 ENM grew again, by another 57% (see Fig. 1).

#### Brokers from All Walks of Life

ENM’s space of intergenerational encounter has been critical to forming new cadre and laying solid groundwork for young women’s interest in feminist activism, but it has not been the only factor. From a strategically positioned intermediate generation have come mediating brokers with an extraordinary capacity of bringing together women from all walks of life. By brokers, we refer to those actors that link otherwise unconnected social groups and people. They are more powerful if situated close to a movement’s “structural holes,” in a position that few people would have, therefore connecting distant social actors that would have an extremely low probability of engaging in social interaction (Burt 2004). More generally, brokers also connect actors across micro and macro social dynamics, with essential functions due to their status (Auyero 2001).

According to our interview data, in Argentina, such brokers, in regular contact with younger people, are university professors and high school teachers, social leaders in grassroots and unemployed workers’ movements (*piqueteros*), union leaders (from teachers’ unions and public sector organizations among others), feminists from left-political parties and the progressive factions of Peronism, as well as those in positions of state decision making. These locations make sense contextually given that in Argentina, political parties and movements are actively rooted in micro-social spaces and daily life of even the poorest urban areas. Historically, they have played an important role in political socialization, and, in the last decade, they have attracted younger participants (Vázquez et al. 2018).

Most of the brokers in our sample are difficult to classify as belonging to only one site. This condition of being multisite residents attests to their capacity to cross social, political and institutional realms and connect diverse women. Although these mediators are not always visible – to the point that one of the experienced feminist interviewees argued that there was a “missing” generation of women between mid-forties and fifties in feminist mobilizations – their commitment to encouraging feminist consciousness and praxis proved decisive for movement massification. These brokers created opportunities for a younger generation while keeping a dialogue with more seasoned feminists.

All the brokers attested to the construction of gender-focused activism within mixed-gender organizations prior to NUM. In more theoretical terms, these brokers “sidestreamed” their approaches and demands into other progressive, often class-based movements and organizations as well as institutions (Alvarez 2014; Borland 2014, 88; Longa 2017). These structures fostered the formation of feminist identities among the youngest activists.

Some brokers focused on bringing together women, particularly those from popular sectors, with micro-organizing and awareness-raising activities that opened spaces for political action. They did not do this alone; they developed a dense network among themselves.^6^ In interviews, they referred to this networking for meaning-making and mobilization “very grassroots militancy,” “very ant-like work, very concrete initiatives,” made possible by strongly committed *compañeras* (solidaristic friends) (broker 4, Peronist political party and feminist collective Mala Junta).

Brokers located in the *piquetero* movements distinguished themselves by recruiting through “women’s assemblies.” The first experiences date back to 2003 and 2004, just after the “Avellaneda massacre.”^7^ This “territorial work” has been a “space for the formation of conscience…opening our eyes and seeing another possible reality” (broker 5, unemployed workers’ movement Frente Popular Darío Santillán, Province of Buenos Aires). Likewise, brokers in progressive unions, who identify with popular and socialist feminism, had been organizing among women in local and neighborhood assemblies prior to the 2015 NUM march.

Our research identified other brokers who were middle-class educated women, namely, professors and researchers who define themselves as “feminists who work and organize inside the university” (broker 6, university faculty and founder of the National Interuniversity Network against Gender Violence). As Crossley (2017) has found for other countries, “college campuses are generative environments for feminism, and valuable sites for the perpetuation of the movement” (148). Beyond peer-to-peer sociality, she makes clear that previous generations’ development and integration of feminist perspectives in curriculum, classrooms, and clubs has played an important role in movement awareness: the “institutional context of higher education teaches the histories of feminists and successes of feminism, thus perpetuating knowledge about the movement” (150). In Ecuador and Peru, Coe (2020) also finds that gender studies programming at the university level stands out as a nexus for intergenerational exchange. It became an “alternative space for political engagement” that was, moreover, somewhat inclusive: developed during a time in which higher education was made available to more than elite students (23).

Our research affirms that the institutional expansion of gender studies as well as interdisciplinary, public programming on women’s health and abortion, enabled professors to inspire young women’s feminism by teaching feminist theory and gender analysis. By the mid- 2000s, as Gogna et al. (2009, 237) show, a younger generation of scholars had developed academic careers that included teaching on gender and sexuality. Students testified to being inspired by feminist teachers who gave them “more tools, like, to express these ideas”; “they open your head: impressive!” One young woman offered the issue of abortion as an example of the impact of these brokers: all she had heard on television was “you are going to kill a person” but going to university “opened the doors for me to a huge number of things that I don’t know if I could’ve gotten to” (young activist 4, university student union, Rosario, Province of Santa Fe). In her view, her feminism blossomed out of her university education:

I think that starting university always marks a lot in people’s lives. At least in my case it was like that. … I am a social work student; I am lucky that all the courses at my school are given from a gender perspective. At the beginning, and I think it happens to all of us a little bit, it’s like ‘I’m in favor of this, but I’m not a feminist, only if I think that women should have the same rights as men, but I don’t I identify myself with the collective’ …. I feel that feminism in my life came from the hand of the university.

Thanks to academic brokers, young women participated in “advanced discussions” on topics often considered taboo. For example, they were fundamental in fostering sex and sexuality education for the younger cohorts, as exemplified by the 2014 “Teachers for the Right to Legal, Safe, and Free Abortion” network that advocated for proper implementation of the Comprehensive Sex Education Law (Gutiérrez 2021, 164).

The specific ambit of gender studies helped in developing an affective politics that those young women refer to as “sorority” with their fellow students and faculty, as they also share and reflect upon their own lived experiences of inequality. In the words of one, intellectual activity came along with a deep sense of belonging to a “community:”

I kind of feel that in all instances of feminist research or learning, some kind of community feeling is always generated that goes beyond academia, which is very good, because they are spaces for exchange that I feel I need and they do me good, and they are also intellectually gratifying. (Young activist 5, local informal feminist collective “Feminismos del Oeste,” Province of Buenos Aires)

Minds and hearts were primed for feminist action in feminist classrooms. And as shown below, even high schools fostered solidaristic linkages leading even younger participants to NUM.

Feminist professors prepared the terrain for mobilizations by centering an analysis of and response to gender violence, whether through inspiring teaching, helping to gather people together at the university, or advancing university protocols to eradicate violence against women on campus.^8^ Before the first NUM, an inter-university network against gender violence had been formed (Vázquez Laba and Rugna 2017). Thus, when the call went out to organize the first NUM in March 2015, these university groups reached out to their campuses, not only urging participation, but also establishing meeting places to generate and sustain a collective presence. Young women referenced preparations for the NUM march carried out at the university premises – such as radio programs, inviting other students, and making posters and songs for the event:

The most important thing was the visits to the classrooms. We …knocked on the door and invited people to attend the march. … We broadcasted an open radio program inside the school; we went out looking for people, we hung posters, and conducted different activities that anyone who passed by noticed or caught their attention. But with this passing through the classrooms, we made our fellow students listen to us. (Young activist 7, university student union, Rosario, Province of Santa Fe)

In short, the intermediate generations of brokers fostered the organizational and personal ties that could bring young women together and connect them with more experienced activists.

Thus, by the time of NUM, spaces for feminist learning and feminist brokers had contributed to a seachange in intergenerational tensions over feminism. Despite differences in organizational style, priorities, and identities, experiences in the unique feminist space of the ENM and multi-class emancipatory mentoring played key roles in what became visible with NUM: feminism as a mass movement. We now turn to analyzing how the specific ideas, organization, and actions of NUM contributed to attracting young women.

### NUM’s Progressive Style

In the case of NUM, how the journalists and artists of this wider and versatile group crafted and distributed mobilizational messages before, during and after the first mass rally, especially through digital formats, proved vital for youth recruitment. They also were active in constructing the “discourse of movement and logistics” (Sutton 2020, 10) attuned to ideas and strategies that would resonate with young women. As a result, the NUM movement adopted a deliberately open structure, characterized by liberating ideas and repertoires of action, thus resonating with the generational style of progressive youth. We organize our analysis following the three main features of a progressive youth style, as delineated in Table 1: emancipatory framework, fluid movement structure, and performative repertoires.

#### Emancipatory Framing

Members of the original NUM collective were particularly skillful at designing visible, appealing and urgent demands and disseminating them through intertwined on- and off-line strategies (Gago 2018). These organizers relied on both professional and personal social networking to call for the original march, making digital media fundamental to NUM’s creation and explosive expansion. The underlying claim of NUM was that violence against women was a form of (cruel) disciplinary regulation over their freedom, by inflicting pain on their bodies to the point of killing them. “Patriarchy,” as NUM members state, “mutilated” women’s lives, made palpable with the extreme form of feminicide of very young women.

The interpretive work that the collective disseminated over social networks started with the slogan “Ni Una Menos” itself. They were inspired by the Mexican poet Susana Chávez’s famous phrase “Ni Una Muerte Más” (Not One More Death), written in response to the notorious feminicides in Ciudad Juárez (Monárrez Fragoso 2002). But the group decided to replace a death-centered claim with a life-oriented demand, a rhetorical move attuned to young people. Based on their insistence that society should not tolerate any more feminicides, they asserted the need to actively prevent it. Indeed, a NUM organizer recalled the agency they attempted to evoke through the slogan:

Ni Una Menos is ‘you don’t get one more out of me.’ It seems … very subtle, but we live from that subtlety… ‘Ni Una Más’ is re-victimizing, it is counting the dead and we count the dead, we remember them, we carry them with us, we turn them into a banner; but more than being on the defensive, we are angry and wanting to produce our own meanings, because we are together. (NUM organizer 2, journalist affliated with the Madres de Plaza de Mayo)

This imagery converged with young women’s concerns around autonomy and self-fulfillment, against old-fashioned, conservative, and violent social arrangements.

Similarly attuned to youth concerns, a distinctive feature of NUM messages was the sense of urgency. The fact that the feminicides that most directly inspired NUM were of teenaged girls made the subjective meaning of time more pressing: “‘Not One Less’ was a phrase that arose out of desperation, as very graphic, like ‘the girls are being taken away from us one by one’” (NUM organizer 3, journalist at the Centro de Estudios Legales y Sociales). This sense of urgency resonated with youth because they feared for their own and their friends’ lives; they could not tolerate delay between actions and goals.

The transformation of NUM’s framework of understanding over time, from initially centered on gender violence and feminicide to later advancing a broader critique of patriarchy and capitalism, kept youth interested and active. As a central example, by the first anniversary of the original protest, NUM’s intersectional social justice frame expanded to include abortion rights by focusing on the dangers of imprisonment, harm, and even death due to illegal procedures particularly for poor women (Daby and Moseley 2021, 27–31). These broader demands resonate with youth culture and values, such as emancipation from all forms of necessity, whether anchored in gender discrimination, precarious life conditions, or traditional social conventions.

This thematic expansion was welcomed by younger feminists, many of whom start from intersectional perspectives and often label themselves “feminists and transfeminists (*transfeministas*).” They believe that all women, cis and trans, and trans men, and other gender “dissidents,” as they are known in Argentina, can be feminists, because, as one explained: “transfeminism understands that the patriarchal system not only oppresses based on gender but also controls and limits all bodies to, in one way or another, adapt them to the established social order” (young activist 11, in university student networks, San Martín, Province of Buenos Aires). They think the earlier women’s movement initially “had a huge impact…making a lot of violences visible and put issues and themes that weren’t talked about in society on the agenda, but that is not the extent of the violences that a trans person, people who aren’t cis men, suffer, to say it that way, more broadly. Thus, for me to claim this name calls attention and an invitation to the struggle to all of those *compañeres* …” (Young activist 9, center-left political party, Rosario, Province of Santa Fe). The expansion of NUM demands and frameworks dovetailed with younger participants’ capacious understanding of feminism, keeping them active in the protest cycle ahead.

#### Fluid Movement Structure and Horizontal Networking

NUM emerged through a flexible movement structure, as an ever-changing flow beyond conventional political channels, open to various types of adherents, and with a horizontal and digitally facilitated network structure attractive to a younger generation. Also, the fact that the NUM group was not clearly inscribed in a political party appealed to young people who themselves had a range of ideological viewpoints or were disconnected from traditional politics. Taking the off- and online platforms, their uses and users together, NUM’s flexible organizational infrastructure enabled connection within and across generations, as well as debate that nurtured feminist exchange and a cycle of protest.

NUM’s lack of hierarchies and gatekeeping meant that any group could affiliate and participate in the demonstration. The network-like form of NUM, with communal leadership, no entry barriers, and porous borders, proved very open to a new generation of activists. As evidenced in their generational style, young women do not necessarily organize around discrete collectives (though some do). They shift activist sites and build more sporadic collectives that constantly give rise to new ones (González Oviedo 2018). Younger protesters saw that it was enough to identify with NUM’s claims, post the slogans and icons on social media, and join the street protest with their friends.

Young women’s reliance on flexible organizational boundaries clearly drew on their experiences in the active student movement of the time.^9^ When the call arose for NUM, many young women had already been mobilized around student issues, demanding less hierarchical educational relationships as well as adding sex education to the curriculum. Young interviewees associated the massive presence of high school youth at NUM with the vitality of the student movement. The movement fostered linkages among students and teachers as well as among peers, resulting in a “very strong bond” of trust and confidence, as the following young activist conveys:

At that time, it was not only the women’s movement, but it also intersected with the student movement, which had had a very strong weight in secondary schools and accompanied the university movement. So, at that time I participated a lot in the student center of my high school, and I remember that we activated a lot with my classmates and even with teachers, and that we built a very strong bond between teachers and students for this slogan of Ni Una Menos, and even taking it out of the classroom and to the streets. (Young activist 8, high school student union, City of Buenos Aires)

As suggested by the work on micromobilization, school-based networks supported younger participants’ experiences and engagement.

NUM thrived on the peer-to-peer socialization of such networks. *Compañeras* abound in younger participants’ narratives, whether the high school friends who used their Instagram feeds full of handmade protest signs to urge each other to go to the huge first march; the college classmates sharing favorite feminist theory texts; or the protest banner painters in student assemblies. As one activist mused, “the reality is that the way I’ve been a feminist activist was [in] a space of activist construction with my *compañeras*.” Drawn together by their personal realization that they were at risk for their gender, whether from personal experiences or through the media coverage of the murder of teenage girls, they founded teen girl organizations, then went into larger student spaces or even testified before the Minister of Education, insisting on the protection of their rights at school. Ultimately, as one said, “feminism is…to construct ourselves through our friendships and our friends” (young feminist 8, high school student union, City of Buenos Aires). They and their friends extended their repertoire to participation in the NUM assemblies, which were open to their intersectional activism (Gago 2019).

Digital media undergirded the network form, and served to create NUM nodes across the country, because the hashtag inspired many groups in different localities. In all likelihood, the scale of the protest would not have been possible without social networks. As one organizer explained, social media was not just a tool, but another space for mobilization. She attested to the intermingling of virtual and physical spaces which together enabled the mass scope:

[We] considered the networks as one more space, not as a medium that is just a post: you occupy the streets, you go to Congress, and you have to occupy the networks too … There were even requests that people from the provinces send their photos of the marches so we were uploading it almost instantly, even their flyers from before the marches, but also of the crowded squares and the actions that had been taken, so it was an instrument of federalization and network. … It is a space of relationship, of relationship and network with people who think similarly and feel that you are not alone, and at the same time they also know where to find you. (NUM organizer 4, performance studies scholar)

Digitally enhanced communication styles related to the cultural expressions of the youth, who were even more inclined to use these means in the micropolitics of interaction than more seasoned feminists. As the following young activist points out, posters and digital images helped to turn them out in the streets:

Well, I found out about the first NUM from my friends, because I remember that I was watching what was happening on social networks and I saw that everyone was posting … I saw that my friends were going, that they had posters and there I saw what was happening and, later, [I went with people from] my university. (Young activist 9, center-left political party, Rosario, Province of Santa Fe)

For this generation, social media provides a communicational infrastructure through which they can not only grow in awareness but also become content protagonists.

#### Performative Repertoire

NUM’s expressive protest repertoire proved central to attracting young people. With the ideas of freedom and emancipation from patriarchal violence as the backdrop, the movement allowed for a new, or newly reconfigured, arsenal of prefigurative cultural practices and performances in the demonstrations. As a highly public movement, it deepened young women’s feminist commitments and identities through widespread embodied artistic expression.

The NUM protest, itself, was an instance of sense making and identity formation through publicly sharing different forms of subjective expression. Performative actions spanned a range of expressions: the handmade posters that alluded to the hashtag or slogans, artistic groups performing live on the streets and sidewalks, half-naked women adorned with body paint, musicians playing their instruments and singing. Specifically, the body politics manifested in NUM ranged from expressions of sexual freedom and choice (kiss-ins), desexualization of women’s bodies (going bare breasted), and the artistic commemoration of murdered women (painting silhouettes of dead bodies), among others. At the rally, people came with their own handwritten posters, telling their personal stories. These stimulated self- and communal reflection, as expressed by a young participant who was a political party activist at the moment of NUM:

Even in that first march, some *compañeras* saw glitter, or a [bare-breasted] girl with the word ‘whore’ written on her forehead. Some *compañeras* said how we should name ourselves like that, that we are self-discriminating. That discussion helped us to think about why ‘whore’ is an insult, what the role it has in society... We met in the street with all our differences, considering what role do we want, what is the feminist agenda that we want and that it is intersectional. (Young activist 10, Peronist political party, Frente de Mujeres del Movimiento Evita de Morón, Province of Buenos Aires)

These actions led to a moment of collective creativity where different cultural expressions converged, generating forms of bricolage, another key aspect of youth style.

A young woman who started her activism at the 2015 NUM described how preparing for these performative actions also became a shared ritual: “You saw that the marches now go with glitter, or we sing our own things or we invent ways to recognize each other, perhaps, within a march: we tie a ribbon around our arms, it’s like something more similar to a ritual” (young activist 7, university student union, Rosario, Province of Santa Fe). As Sutton (2020) identified in her exploration of intergenerational collaboration in Argentine abortion rights protests, the *pibas’* energy sustained movement, particularly through street actions and other physical demonstrations, and festive body-focused activist practices to physically embody demands for love, freedom, and autonomy.

As with other elements of movement organization, collective creativity was also supported through digital media, which became intrinsic to the cultural work of meaning construction. Digital interactions inspired off-line activities (make a banner, draw a poster, meet with other people) that in turn shaped virtual messages and images. Digitally enhanced activism within an artistic venue generated empathy and spread anger about and intolerance of the murder of young women. In short, the rituals of NUM protest repertoires, enhanced by digital resources, helped young participants realize their feminist identities through collective experiences and engage in a mass movement.

## Conclusion

Ni Una Menos has its own distinct chapter in the history of Argentine and Latin American feminism. Innovating social movement repertoires, NUM disrupted the public space with new cultural messages and witnessed the rise of young feminism and transfeminism as a privileged political subject for the years to come. Although this movement was intimately linked to a particular context, our research sheds light on critical factors that account for movement intergenerational cooperation through dialogue and trust building.

As a case of movement renewal through rejuvenation rather than demographic replacement, NUM calls for a more nuanced explanation of why feminists from distinct cohorts may converge in joint mobilization. Such processes can be obscured by analytic approaches that are based on assumptions of generational replacement within movements. Against the idea of separate “waves,” or even spin-offs, our research advances the concept of productive mediation, as we have uncovered conditions that allow women from different generational units to act towards common goals. Rather than disidentification, conflict, and competition, we identify social processes of learning and exchange, as well as affective bounds of sorority. To fully understand this, we emphasize the importance of networks and networkers, as well as how movement messages, organization, and repertoires appeal to young participants.

To explain NUM’s success, we identified long-term conditions including ongoing spaces for encounter as well as the dedicated work of “brokers” from an in-between generation of social activists, union and political leaders, and gender studies professors identified with feminism. Argentina has a long tradition of fostering the social infrastructure of feminist encounters that, eventually, laid a solid foundation upon which several feminist generations met. Brokers created communities of learning and understanding that helped attract and mobilize young and very young women. Their persistent cultural and organizing work offered feminist structures of opportunities in unions, universities and high schools, social movements, and other settings, that brought young people into NUM. These profuse networks at the micro-social level also fostered peer interactions, as friends and student groups pulled in young people. Activists found pathways through a host of supportive spaces, from high school and university settings to other political organizations, to the excitement of the yearly National Women’s Encounter. The NUM collective catalyzed these conditions into mass intergenerational mobilization. Their life-centered framework demanding an end to feminicide expanded to include a radical critique of capitalism alongside patriarchy, and an intersectional approach which dovetailed with young people’s understanding of feminism and transfeminism. NUM’s lack of hierarchies and movement gatekeeping facilitated young people’s multiple connections, bound by fluid ties reinforced by digital connections as their preferred form of communicating and gathering. Finally, the expressive nature of the social protest itself was an instance of ritualized sense-making and identity formation through the kinds of bodily, performative actions particularly appealing to youth.

With these findings, we argue for a meso-level approach to analyzing social movement development. Jo Reger (2012) identified an inverse relationship between the relative openness of a given political context and the degree of intergenerational conflict in feminist movements. We, however, focused our analytic lens on how feminist of all walks of life, and ages, constructed a range of networks that allowed for a mass movement to emerge. By giving agency to movement participants, we show that, beyond context, we must take into account networks, networkers, and frameworks.

At the most general level, our work demonstrates that capturing the complexity of movement rejuvenation requires a conceptual shift to accommodate the fact that, at present, different generations of feminists are apparent in the same movement space. Mass social movements that take to the streets during sustained cycles of protests are communities of learning with solidarity ties that bind members together and, as such, are able to accommodate, if not combine, generational styles. The successful mass progressive movements of the future may well be predicated on not only building strong foundations for welcoming the next generations to the revolution, but also at some level catering to their strong preferences for decentralized, performative, diverse, digitally enhanced opportunities for meaning making and social impact.

## Footnotes

1 Although we refer to these actors as “women” throughout the piece to reflect their dominant gender identity, gender-non-binary, gender-queer, and male-identified participants have also taken part in this mobilization.

2 It is really no wonder students are past masters at activating the social networks that enable the spread of ideas and foster collective cooperation, given their use of social media and many opportunities for face-to-face sociality. Their classes can also act as important spaces for communal contemplation of contemporary conflicts.

3 This section draws on material discussed in Friedman and Rodríguez Gustá 2023

4 The “Madres de Plaza de Mayo” is a central organization in the human rights movement that fought against the dictatorship in Argentina. They are the mothers whose children (mostly young and very adults) disappeared, tortured and executed by the military regime. Founded in 1977, they have maintained a walk around Plaza de Mayo (the square in front of the presidential palace) every Thursday wearing a white scarf as a symbol of their claims for justice and democracy.

5 Reflecting their commitment to diversity and inclusion, participants renamed the ENM the Plurinational Encounter of Women and Dissidents, Lesbians, Travestis, Trans, Bisexuals, Intersexuals and Non-Binaries in 2021. We use the original name to reflect what was used by our interviewees, who were referring to participation prior to 2021.

6 As their interviews reveal, brokers’ ability to maintain these micro-spaces demonstrates their network construction capabilities, such as with professionals for the right to decide, and the pre- and post-abortion counseling services, which makes it possible to invite more marginalized women to participate in feminist reflections. In municipal management positions, these brokers promoted the organization and mobilization of women on a neighborhood scale.

7 The “massacre” refers to the murder of two social activists, Máximo Kosteki and Darío Santillán, and the following protest events and repression that took place on June 26, 2002 in the town of Avellaneda in the Province of Buenos Aires.

8 Starting in 2009, with the passage of Law 26,486 on Comprehensive Protection to Prevent, Punish and Eradicate Violence against Women, national universities began to move forward with action protocols for cases that occurred on their premises. By the mid-2010s, some had specific programs to address gender-based violence, including awareness-raising activities in college classrooms.

9 In 2012, for example, there were tomas (seizures) in 29 Buenos Aires schools, reaching 28,000 young people. By the time of NUM in June 2015, students had been protesting and occupying schools since the beginning of the academic year in March.

## References

[CR1] Alcaraz, María Florencia, and Agustina Paz Frontera. 2018. La generación “Ni Una Menos.” In *El Atlas de la revolución de las mujeres. Las luchas históricas y los desafíos actuales del feminismo*, eds. Creusa Muñoz, Luciana Garbarino, and Laura Oszust, 30–33. Buenos Aires: Capital Intelectual.

[CR2] Alvarez Sonia E (2014). Para além da sociedade civil: Reflexões sobre o campo feminista. Cadernos Pagu.

[CR3] Alma Amanda, Lorenzo Paula (2013). Mujeres que se encuentran. Una recuperación histórica de los Encuentros Nacionales de Mujeres en Argentina (1986–2005) (Women who meet. A historical recovery of the National Women's Encounters in Argentina (1986–2005)).

[CR4] Auyero Javier (2001). La política de los pobres. Las prácticas clientelistas del Peronismo (The politics of the poor. The clientelist practices of Peronism).

[CR5] Borland, Elizabeth. 2014. Dynamics and identity: Conflict and cooperation among feminists in Buenos Aires. In *Intersectionality and social change*, ed. Jessica L. Beyer and Lynne M. Woehrle, 83–106. Bingley: Emerald Group Publishing. 10.1108/S0163-786X20140000037003.

[CR6] Borland Elizabeth, Sutton Barbara (2007). Quotidian disruption and women’s activism in times of crisis, Argentina 2002–2003. Gender and Society.

[CR7] Burt Ronald S (2004). Holes and good ideas. American Journal of Sociology.

[CR8] Burton, Julia. 2019. *A la nequina. Un estudio sobre la militancia feminista no metropolitana por el derecho al aborto*. Tesis para obtener el grado de Doctora en Sociología, Universidad Nacional de San Martín.

[CR9] Caren Neal, Raj Andrew Ghoshal, Ribas Vanesa (2011). A social movement generation: Cohort and period trends in protest attendance and petition signing. American Sociological Review.

[CR10] Castro Luis (2018). La acción colectiva feminista, ¿de la lucha de clases a la lucha de géneros? Apuntes para la comprensión/práctica de los movimientos sociales, en torno al caso “Ni Una Menos.". Ciencia Política.

[CR11] Chamberlain Prudence (2017). The feminist fourth wave: Affective temporality.

[CR12] Cochrane Kira (2013). All the rebel women: The rise of the fourth wave of feminism.

[CR13] Coe Anna-Britt (2020). Social processes underlying movement influence: Young adult feminist activists’ interactions with professionalized feminist organizations in Ecuador and Peru. The Sociological Quarterly.

[CR14] Coe Anna-Britt, Vandegrift Darcie (2015). Youth politics and culture in contemporary Latin America: A review. Latin American Politics and Society.

[CR15] Chironi, Daniela. 2019. Generations in the feminist and LGBT movements in Italy: The case of Non Una Di Meno. *American Behavioral Scientist* 63(10):1–28. 10.1177/0002764219831745

[CR16] Crossley Alison Dahl (2017). Finding feminism: Millennial activists and the unfinished gender revolution.

[CR17] Crossley Nick (2008). Social networks and student activism: On the politicising effect of campus connections. The Sociological Review.

[CR18] Crossley, Nick, and Mario Diani. 2018. Networks and fields. In *The Wiley Blackwell companion to social movements*, eds. David. A. Snow, Sarah. A. Soule, Hanspeter Kriesi, and Holly J. McCammon, 149 – 66. Hoboken: Wiley Online Library.

[CR19] Cullen Pauline, Fischer Clara (2014). Conceputalising generational dynamics in feminist movements: Political generations, waves and affective economies. Sociology Compass.

[CR20] Daby Mariela, Moseley Mason W (2021). Feminist mobilization and the abortion debate in Latin America: Lessons from Argentina. Politics & Gender.

[CR21] della Porta Donatella (2019). Deconstructing generations in movements: Introduction. American Behavioral Scientist.

[CR22] della Porta Donatella (2019). Desconstructing generations: Concluding remarks. American Behavioral Scientist.

[CR23] della Porta, Donatella. 2014. In-depth interviews. In *Methodological Practices in Social Movement Research*, ed. Donatella della Porta, 228–261. Oxford: Oxford University Press.

[CR24] Di Marco Graciela (2011). El pueblo feminista: Movimientos sociales y lucha de las mujeres en torno a la ciudadanía (The feminist people: Social movements and women’s struggle for citizenship).

[CR25] Friedman Elisabeth Jay (2017). Interpreting the internet: Feminist and queer counterpublics in Latin America.

[CR26] Friedman Elisabeth Jay, Rodríguez Gustá Ana Laura (2023). “El viento arrollador”: La irrupción de las jóvenes en la protesta del ni una menos de Argentina. Perfiles Latinoamericanos.

[CR27] Gago Verónica (2019). La potencia feminista: O el deseo de cambiarlo todo (Feminist power: Or the desire to change everything).

[CR28] Gago Verónica (2018). La tierra tiembla. Critical Times.

[CR29] George Alexander L, Bennett Andrew (2005). Case studies and theory development in the social sciences.

[CR30] Gogna Mónica, Pecheny Mario, Jones Daniel (2009). Teaching gender and sexuality at public universities in Argentina. International Journal of Sexual Health.

[CR31] González Oviedo, Cintia Andrea. 2018. *Activismo feminista en internet y su impacto en la ciudadanía de las mujeres*. Unpublished manuscript. Buenos Aires: FLACSO PRIGEPP.

[CR32] Goodwin Jeff, Jasper James M, Polletta Francesca, Goodwin Jeff, Jasper James M, Polletta Francesca (2001). Why emotions matter. Passionate politics. Emotions and social movements.

[CR33] Gordon Hava R (2007). Allies within and without: How adolescent activists conceptualize ageism and navigate adult power in youth social movements. Journal of Contemporary Ethnography.

[CR34] Gordon Hava R, Taft Jessica K (2011). Rethinking youth political socialization: Teenage activists talk back. Youth & Society.

[CR35] Goss Kristin A (2013). The paradox of gender equality: How American women’s groups gained and lost their public voice.

[CR36] Gutiérrez María Alicia (2021). Rights and social structure. The experience of the National Campaign for the right to Legal, Safe, and free abortion in Argentina. Abortion and democracy. Contentious body politics in Argentina, Chile, and Uruguay.

[CR37] Henry Astrid (2004). Not my mother’s sister: Generational conflict and third wave feminism.

[CR38] Hewitt Nancy A, Hewitt Nancy A (2010). Introduction. No permanent waves: Recasting histories of U.S. feminism.

[CR39] Hirsch Eric L (1990). Sacrifice for the cause: Group processes, recruitment, and commitment in a student social movement. American Sociological Review.

[CR40] Jasper James (1998). The emotions of protest: Affective and reactive emotions in and around social movements. Sociological Forum.

[CR41] Jasper James (2011). Emotions and social movements: Twenty years of theory and research. Annual Review of Sociology.

[CR42] Juris Jeffrey Scott, Pleyers Geoffrey Henri (2009). Alter-activism: Emerging cultures of participation among young global justice activists. Journal of Youth Studies.

[CR43] Lamas Marta (2021). Dolor y política. Sentir, pensar y hablar desde el feminismo (Pain and politics. Feel, think and speak from feminism).

[CR44] Larrondo, Marina, and Camila Ponce Lara. 2019. Activismos feministas jóvenes en América Latina. Dimensiones y perspectivas conceptuales. In *Activismos feministas jóvenes: Emergencias, actrices y luchas en América Latina*, eds. Marina Larrondo and Camila Ponce Lara, 21–40. Buenos Aires: CLACSO.

[CR45] Laudano Claudia, Chaher Sandra (2018). Acerca de la apropiación feminista de TICs. Argentina: Medios de comunicación y género ¿hemos cumplido con la plataforma de acción de Beijing?.

[CR46] Longa, Francisco. 2017. Del antipatriarcado al feminismo: Derivas del ethos militante en un movimiento social de la Argentina (2004–2015). *Revista Interdisciplinaria de Estudios de Género de El Colegio de México* 3 (5): 57–89. 10.24201/eg.v3i5.96.

[CR47] López Maria Pia (2020). Not one less: Mourning, disobedience and desire.

[CR48] MacLeavy, Julie, Maria Fannin, and Wendy Larner. 2021. Feminism and futurity: Geographies of resistance, resilience and reworking. *Progress in Human Geography* 45 (6): 1558–1579. 10.1177/03091325211003327.

[CR49] Maher Thomas V, Earl Jennifer (2019). Barrier or booster? Digital media, social networks, and youth micromobilization. Sociological Perspectives.

[CR50] Mann Susan Archer, Huffman Douglas J (2005). The decentering of second wave feminism and the rise of the third wave. Science & Society.

[CR51] Mannheim Karl, Kecskemeti Paul (1952). The problem of generations. Karl Mannheim: Essays.

[CR52] Manzano, Valeria. 2019. Feminismo y juventud en la Argentina del siglo XX. In *Activismos feministas jóvenes: Emergencias, actrices y luchas en América Latina*, ed. Marina Larrondo and Camila Ponce Lara, 41–58. Buenos Aires: CLACSO.

[CR53] Masson Laura (2007). Feministas en todas partes. Una etnografía de espacios y narrativas feministas en Argentina (Feminists everywhere. An ethnography of feminist spaces and narratives in Argentina).

[CR54] Molyneux Maxine, Dey Adrija, Gatto Malu A. C., Rowden Holly (2020). Feminist activism 25 years after Beijing. Gender & Development.

[CR55] Monárrez Fragoso Julia (2002). Feminicidio sexual serial en ciudad Juárez 1993–2001. Debate Feminista.

[CR56] Natalucci Ana, Rey Julieta (2018). ¿Una nueva oleada feminista? Agendas de género, repertorios de acción y colectivos de mujeres (Argentina, 2015–2018). Estudios Políticos y Estratégicos.

[CR57] Osler Audrey, Starkey Hugh (2003). Learning for cosmopolitan citizenship: Theoretical debates and young people’s experiences. Educational Review.

[CR58] Pisetta, Antonella. 2019. ¿Cómo surgió el movimiento Ni Una Menos?. *Perfil*, March 7, 2019. https://www.perfil.com/noticias/sociedad/como-surgio-movimiento-ni-una-menos-2015.phtml. Accessed 29 June 2020.

[CR59] Ponce Lara Camila (2020). El movimiento feminista estudiantil chileno de 2018: Continuidades y rupturas entre feminismos y olas globales. Izquierdas.

[CR60] Portos Martin (2019). Divided we stand, (oftentimes) united we fight: Generational bridging in Spain’s feminist movement and the cycle of antiausterity mobilizations. American Behavioral Scientist.

[CR61] Ragin Charles, Ragin Charles C, Becker Howard S (1992). Introduction: Cases of “What is a case?”. What is a case? Exploring the foundations of social inquiry.

[CR62] Reger Jo (2012). Everywhere and nowhere: Contemporary feminism in the United States.

[CR63] Reguillo Cruz Rossana (2017). Paisajes insurrectos. Jóvenes, redes y revueltas en el otoño civilizatorio (Insurrectionary landscapes. Youth, networks and revolts in the civilizational autumn).

[CR64] Safa Helen Icken (1990). Women’s social movements in Latin America. Gender and Society.

[CR65] Santoro, Sonia. 2019. Empieza el 34 Encuentro Nacional de Mujeres. *Página 12* https://www.pagina12.com.ar/224830-empieza-el-34-encuentro-nacional-de-mujeres. Accessed 29 June 2020.

[CR66] Snow David A, Burke Rochford E, Benford Robert K (1986). Frame alignment processes, micromobilization, and movement participation. American Sociological Review.

[CR67] Snyder Cara K, Wolff Cristina Scheibe (2019). The perfect misogynist storm and the electromagnetic shape of feminism: Weathering Brazil’s political crisis. Journal of International Women’s Studies.

[CR68] Strauss Anselm, Corbin Juliet (2002). Bases de la investigación cualitativa. Técnicas y procedimientos para desarrollar la teoría fundamentada (bases of qualitative research. Techniques and procedures for developing grounded theory).

[CR69] Sutton Barbara (2020). Intergenerational encounters in the struggle for abortion rights in Argentina. Women’s Studies International Forum.

[CR70] Tabbush Constanza, Caminotti Mariana (2015). Igualdad de género y movimientos sociales en la Argentina posneoliberal: La organización barrial Tupac Amaru. Perfiles Latinoamericanos.

[CR71] Taft Jessica K (2011). Rebel girls. Youth activism and social change across the Americas.

[CR72] Tavory Iddo (2020). Interviews and inference: Making sense of interview data in qualitative research. Qualitative Sociology.

[CR73] Vázquez Laba Vanesa, Rugna Cecilia (2017). Acción colectiva en torno a la agenda feminista sobre violencia de género en las universidades nacionales argentinas. Boletín Científico Sapiens Research.

[CR74] Vázquez Melina, Rivarola Dolroes Rocca, Cozachcow Alejandro (2018). Compromisos militantes en juventudes político-partidarias (Argentina, 2013–2015). Revista Mexicana de Sociología.

[CR75] Ward Matthew (2016). Rethinking social movement micromobilization: Multi-stage theory and the role of social ties. Current Sociology Review.

[CR76] Whittier, Nancy. 1995. Turning it over: Personnel changes in the Columbus, Ohio, women’s movement, 1969–1984. In *Feminist organizations: Harvest of the new women’s movement*, eds. Myra Marx Ferree and Patricia Yancey Martin, 180–198. Philadelphia: Temple University Press.

[CR77] Whittier Nancy (1997). Political generations, micro-cohorts, and the transformation of social movements. American Sociological Review.

[CR78] Whittier, Nancy. 2004. The consequences of social movements for each other. In *The Blackwell companion to social movements*, eds. David A. Snow, Sarah. A. Soule, and Hanspeter Kriesi, 531–551. Oxford: Blackwell Publishing LTD.

